# Evaluation of anxiolytic-like activity of *Vitis vinifera* juice in mice

**Published:** 2016

**Authors:** Muhammad Aslam, Nuzhat Sultana

**Affiliations:** Department of Pharmacology, Faculty of Pharmacy, University of Karachi-75270, Pakistan

**Keywords:** *Anxiety*, *Flavonoids*, *Open field test*, *The light/dark box test*, *Vitis vinifera*

## Abstract

**Objective:**

Scientific studies have shown that* Vitis vinifera* (*V. vinifera*) contains flavonoids and stillbenoids. Flavonoids are well known to possess anxiolytic activities. In view of the idea that flavonoids present in *V. vinifera* could be useful in anxiety, we evaluated anxiolytic-like activity of *V. vinifera* juice (VVJ).

**Materials and Methods:**

Light/dark box and the open field test were used to assess the anxiolytic potential of *V. vinifera* juice (VVJ). The juice was given orally by gavage at the dose of 4 and 8 mL/kg body weight. Diazepam (1 mg/kg i.p.) was used as the standard drug.

**Results:**

It was observed that the juice produced significant and dose dependent increase in the time spent in light cubicle (p<0.001), transfer latency from the light to dark cubicle (p<0.001) and the number of transitions between the two cubicles (p<0.001) as compared with the control group. *V. vinifera* also demonstrated significant and dose dependent increase in ambulation (P<0.001) and rearing (p<0.001) in open field test as compared to the control group.

**Conclusion:**

In conclusion, the present study establishes the anxiolytic-like activity of VVJ in animal models of anxiety.

## Introduction

Anxiety is characterized as "A feeling of apprehension, uncertainty or tension stemming from the anticipation of an imaginary or unreal threat" (Khan et al., 2011). Studies have demonstrated that one-eighth of the aggregate population of the world has been influenced by anxiety. Anxiety has turned into an essential zone of research for psychopharmacologists amid this decade (Yadav et al., 2008). Excessive anxiety can have a harmful impact on the quality of life (Seo et al., 2007). Benzodiazepines, GABA_A_ receptor agonists, buspirone and 5-HT_1A _receptor agonists are the standard medications used to treat anxiety (Lader & Morton, 1991). These standard medications have been connected with various undesirable reactions, for example, muscle relaxation, dizziness, sedation, physical reliance, memory aggravation, headache, nervousness, paresthesia, diarrhoea, excitation and sweating and drug interactions (Jordan et al., 1996; Alan, 2000). Therefore, there has been considerable interest in the exploration of new anxiolytic medications with fewer side effects (Holm, 1988; Balinger, 1990). New synthetic drugs as well as herbal drugs may have a conceivable remedial importance in the treatment of anxiety (Beaubru and Gray, 2000). Herbal medications are the ancient form of health care known to human beings (Muhammad and Syed, 2013). These medications are claimed to be more secure ones because of the fact that they have demonstrated few of the unfavorable medication responses when contrasted with new synthetic drugs (Biren and Avinash, 2010). *Vitis vinifera* (*V. vinifera*) Linn (grapes) family vitaceae also called angoor (Urdu) is an inexplicable herb containing various dynamic phytochemicals of pharmacological noteworthiness. The oldest records of the use of grape products by humans date back to 3500–2900 B.C (Bowers et al., 1999). Among the *Vitis* species, *V. vinifera* is the most cultivated fruit crop around the world because of its use in wine production (Lodhi & Reisch, 1995). Research studies demonstrate that *V. vinifera* has antioxidant (Silva et al., 1991), anticancer and antimutagenic activity (Liviero and Puglisi, 1994; Tyagi et al., 2003), cardioprotective activity (Frankel et al., 1993; Frankel et al., 1995), antiulcer activity (Saito et al., 1998), anti-inflammatory activity (Amella et al., 1985), and antiallergic activity (Hansen, 1995).

*V. vinifera* is a rich source of flavonoids and stillbenoids (Kashif et al., 2010), Flavonoids are well known to possess anxiolytic activity (Paladini et al., 1999). To the best of our knowledge, there has been no investigative study to assess the anxiolytic potential of* V. vinifera*. In view of the idea that flavonoids present in *V. vinifera* could be useful in anxiety, we evaluated anxiolytic-like activity of *V. vinifera* using light/dark box and open field test.

## Materials and Methods

**The collection of plant material**

Fresh fruits of *V. vinifera* were purchased from local markets, Karachi, Pakistan. A pharmacognosist of the Department of Pharmacognosy, Ziauddin University, Pakistan, authenticated the sample. Voucher specimen (P/PHL1390) was deposited in the institute for future reference. *V. vinifera* fruits were squeezed by hand in a muslin cloth to yield fresh juice. 

**The selection of animals**

This study was conducted utilizing male Swiss albino mice weighing between 20 to 25 g. The specifications given in Helsinki Resolution 1964 were followed during animal handling. This research was approved by the Board of Advanced Studies and Research, University of Karachi vide BASR resol. No. 16 dated 26-08-2013.

**Dosing**

The dose of the juice was calculated according to the body weight of the mice. The juice was administered in mice at two different doses, i.e., 4 mL/kg and 8 mL/kg. The dosing of the juice was done once daily at 10 a.m. for 60 days.

**Methodology**

A total number of 40 healthy Swiss albino mice weighing between 20 to 25 g were procured from the animal house of University of Karachi, Pakistan. They were kept five per cage in polypropylene cages with a layer of sawdust litter under controlled conditions at room temperature 25-30 °C, relative humidity of 45–55% and 12/12 hours light-dark cycle. The mice were given standard pellets and water *ad libitum*. The pellets and water were withdrawn six hours before the administrations and during the experiments. The mice were divided into four groups *viz*.:

Group I: Normal control, given normal saline 8 mL/kg, p.o.

Group II: Treatment group, given VVJ 4 mL/kg, p.o.

Group III: Treatment group, given VVJ 8 mL/kg, p.o.

Group IV: Positive control, given diazepam 1 mg/kg, i.p. 

**Assessment of anxiolytic activity**

*The light/dark box test (LDBT)*

The apparatus used in this test consisted of an open-top wooden box with two distinct cubicles, namely, a dark safe cubicle and a light aversive cubicle. The dark cubicle was painted black and illuminated with a dim red light. The light cubicle was painted white and brightly illuminated with a 100 W white light source placed 17 cm above the box. The test was commenced by placing a mouse in the centre of the light cubicle. The observed parameters were the time spent in the light cubicle, transfer latency (time taken for the first entry in dark cubicle) and the number of transitions between two cubicles. The parameters were measured for 10 minutes (Muhammad and Rahila, 2013; Khan et al., 2011). The test was conducted on the 7^th^, 15^th^, 30^th^ and 60^th^ day of the study, that is, the animals were examined 4 times during the 60 days of the study.

*Open field test (OFT)*

The apparatus used in this test was consisted of a wooden box (60 × 60 × 30 cm^3^) with the floor divided into 16 squares (15 × 15 cm^2^). The apparatus was illuminated with a 40-W lamp suspended 100 cm above. The test was commenced by placing a mouse in the centre of the field and was left for 2 minutes for acclimatization with the apparatus. The observed parameters were number of squares (central and peripheral) crossed, 

rearings (number of times the mouse stood on its hind limbs) and faecal droppings (number of faecal droppings excreted during the test period). The parameters were measured for 10 minutes (Sethi et al., 2005; Sonovane et al., 2002). The test was conducted on the 7^th^, 15^th^, 30^th^ and 60^th^ day of the study, that is, the animals were examined 4 times during the 60 days of the study.

**Statistical analysis**

The data expressed are mean ± standard error of mean (SEM). Values were compared with control ones by taking the mean of all of them and the significance of difference between the means was determined by one-way ANOVA followed by Newman-Keuls post hoc test. A difference of p<0.05 was considered statistically significant. All statistical analyses were performed using GraphPad Prism version 5.00 for Windows, GraphPad Software, San Diego, CA, USA.

## Results

**The light/dark box test (LDBT)**

The results summarized in Table 1, 2 & 3 reveal that *V vinifera* juice, at the dose of 4 and 8 mL/kg, demonstrated significant and dose dependent increase in the time spent in light cubicle (p<0.001), transfer latency (p<0.001) and number of transitions between the two cubicles (p<0.001) as compared with the control (saline-treated) group. 

Diazepam, as a standard drug, also exhibited significant increase in the time spent in light cubicle, transfer latency and number of transitions between the two cubicles.

**Table 1:** Effect of *V. vinifera* juice on the time spent in the light cubicle in light/dark box test

**Treatment**	**Time spent in light cubicle (Sec.)** **on day 7**	**Time spent in light cubicle (Sec.)** **on day 15**	**Time spent in light cubicle (Sec.)** ** on day 30**	**Time spent in light cubicle (Sec.)** **on day 60**
**Control** **(Saline 8 mL/kg)**	110.50 ± 1.47	109.50 ± 1.57	109.70 ± 1.59	109.60 ± 1.64
***V. vinifera*** ** juice** **4 mL/kg**	119.1 ± 0.45***	119.9 ± 0.88***	125.3 ± 0.71***	130.9 ± 1.52***
***V. vinifera*** ** juice** **8 mL/kg**	122.6 ± 0.83***	119.8 ± 0.59***	127.5 ± 0.98***	134.2 ± 1.59***
**Diazepam** **1 mg/kg**	163.0 ± 0.78***	162.6 ± 0.77***	162.7 ± 0.81***	162.3 ± 0.70***

Number of animals (n) = 10.The values are mean ± SEM.

* p<0.05,

** p<0.01,

*** p<0.001 when compared with the control group (One-way ANOVA followed by Newman-Keuls post hoc test).

**Table 2:** Effect of *V vinifera* juice on transfer latency in light/dark box test

**Treatment**	**Latency (Sec.)** **on day 7**	**Latency (Sec.)** **on day 15**	**Latency (Sec.)** **on day 30**	**Latency (Sec.)** **on day 60**
**Control** **(Saline 8 mL/kg)**	76.6 ± 2.26	76.6 ± 2.38	76.0 ± 1.97	77.1 ± 2.40
***V. vinifera*** ** juice** **4 mL/kg**	84.2 ± 2.09**	89.0 ± 2.25***	90.7 ± 2.45***	88.8 ± 2.64***
***V. vinifera*** ** juice** **8 mL/kg**	87.1 ± 2.13**	91.5 ± 2.26***	93.1 ± 2.40***	91.2 ± 2.69***
**Diazepam** **1 mg/kg**	136.8 ± 1.07***	137.3 ± 0.96***	137.4 ± 1.02***	136.8 ± 0.92***

Number of animals (n) = 10. The values are mean ± SEM.

* p<0.05,

** p<0.01,

*** p<0.001 when compared with the control group (One-way ANOVA followed by Newman-Keuls post hoc test).

**Table 3:** Effect of *V. vinifera* juice on the number of transitions in light-dark box test

**Treatment**	**Transitions** **on day 7**	**Transitions** **on day 15**	**Transitions** **on day 30**	**Transitions** **on day 60**
**Control** **(Saline 8 mL/kg)**	8.4 ± 0.42	8.1 ± 0.40	7.9 ± 0.43	7.8 ± 0.32
***V. vinifera*** ** juice** **4 mL/kg**	12.2 ± 0.38***	12.5 ± 0.30***	12.8 ± 0.38***	12.9 ± 0.34***
***V. vinifera*** ** juice** **8 mL/kg**	16.2 ± 0.41***	16.6 ± 0.49***	16.7 ± 0.63***	17.5 ± 0.34***
**Diazepam** **1 mg/kg**	21.2 ± 0.55***	21.9 ± 0.60***	21.8 ± 0.55***	22.0 ± 0.51***

Number of animals (n) = 10. The values are mean ± SEM.

* p<0.05,

** p<0.01,

*** p<0.001 when compared with the control group (One-way ANOVA followed by Newman-Keuls post hoc test

**Open field test (OFT)**

**Figure 1:**
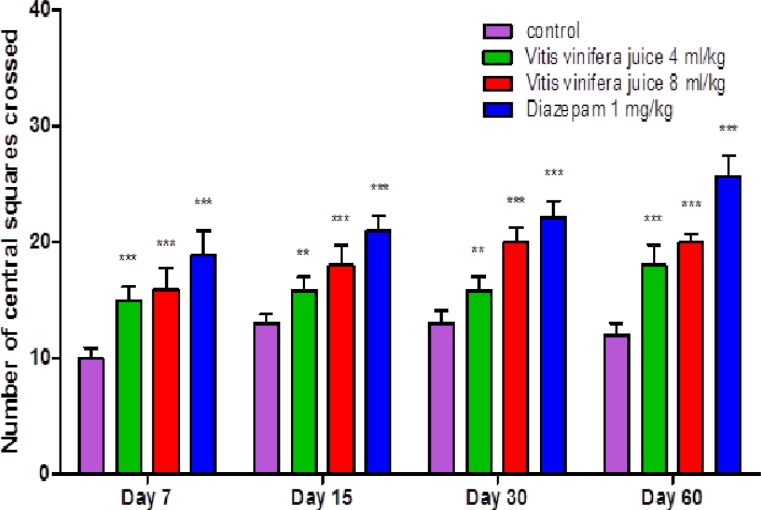
Effect of *V. vinifera* on the number of central squares crossed in open field test. Number of animals (n) = 10.The values are mean ± SEM. *p<0.05, **p<0.01, ***p<0.001 when compared with the control group (One-way ANOVA followed by Newman-Keuls post hoc test

**Figure 2:**
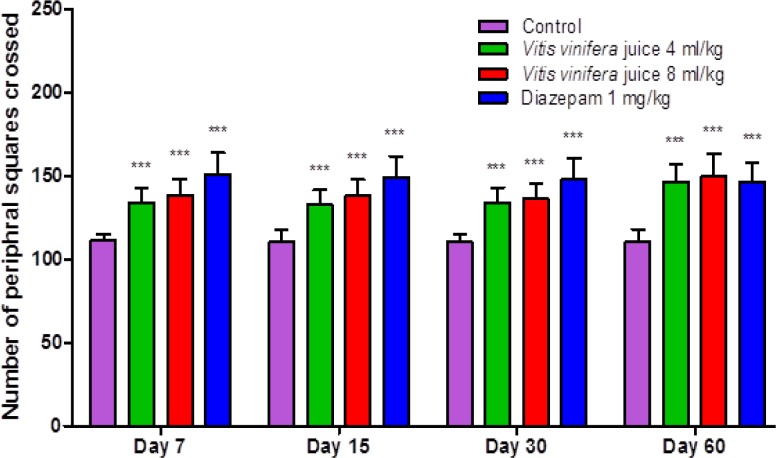
Effect of *V. vinifera* on the number of peripheral squares crossed in open field test.

**Figure 3:**
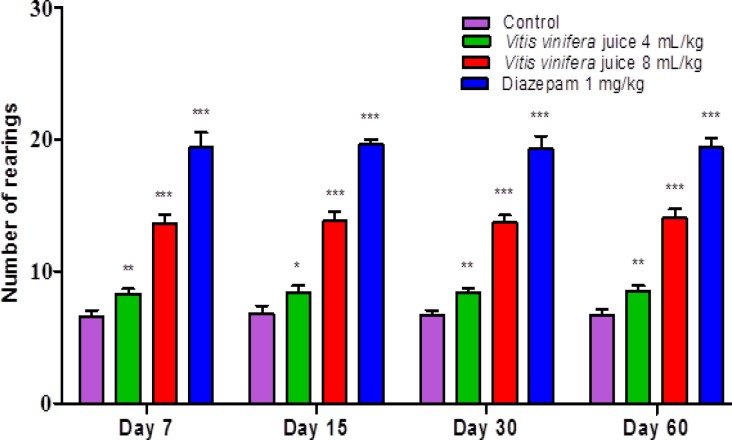
Effect of *V vinifera* on the number of rearings in open field test .Number of animals (n) = 10.The values are mean ± SEM. ^*^p<0.05, ^**^p<0.01, ^***^p<0.001 when compared with the control group (One-way ANOVA followed by Newman-Keuls *post hoc* test

**Figure 4:**
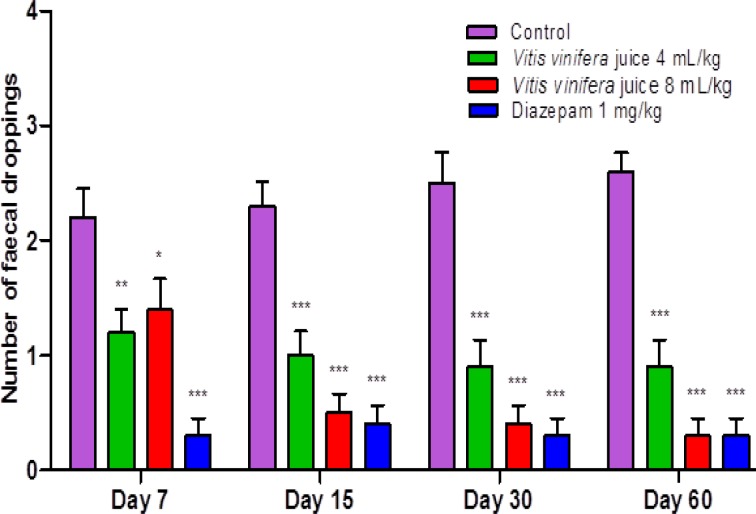
Effect of *V. vinifera* on faecal droppings in open field test. Number of animals (n) = 10. The values are mean ± SEM. *p<0.05, **p<0.01, ***p<0.001 when compared with the control group (One-way ANOVA followed by Newman-Keuls post hoc test

The results summarized in Figures 1, 2, 3 & 4 reveal that *V. vinifera* juice (VVJ), at the dose of 4 and 8 mL/kg, demonstrated significant and dose dependent increase in the number of squares (central and peripheral) crossed and rearing (number of times the animal stood on its hind limbs) and significant decrease in faecal droppings (number of faecal droppings excreted during the test period) in open field test compared with control group. Diazepam also produced significant increase in the number of squares crossed and rearing. Faecal droppings were significantly decreased by diazepam.

Number of animals (n) = 10. The values are mean ± SEM. *p<0.05, **p<0.01, ***p<0.001 when compared with the control group (One-way ANOVA followed by Newman-Keuls post hoc test).

## Discussion

Various animal models are utilized to evaluate the potential anxiolytic activity of different compounds. Diazepam is the most ordinarily utilized standard anxiolytic drug. Diazepam exerts its effects through benzodiazepine (BZ) receptors. Similarly, the drug is also utilized as the standard anxiolytic drug even when the compound under study should not act through benzodiazepine (BZ) receptors (Iyer et al., 2004). 

In the light/dark box test, animals mostly want to pass more time in dark cubicles as contrasted with light cubicles on account of fear of exposure to the novel environment (Belzung et al., 1987). In our study, after administration of VVJ, the mice spent more time in the light cubicle and less time in dark cubicle. Transfer latency from light to dark cubicle was also increased and the number of cubicle entries was likewise increased. These results suggest that VVJ has anxiolytic-like activity. 

The open field test, being a simple, sensitive and reproducible model, is usually utilized for the evaluation of different potential anxiolytic compounds. By the way, the researchers must be extremely watchful that the handling of animals, sound sealing of the framework, and the doses utilized should not influence the motor activity of the animals. As any change in motor activity by parameters other than the drug, can make the test outcomes temperamental. At the point when an animal is presented in a novel environment, the animal faces emotional disturbance, tension and anxiety. In an open field test, the locomotion of an anxious animal is strikingly diminished. The animal also likes to pass more time in the peripheral areas and there is also change in normal rodent behavioural patterns including reduction in rearing and grooming (Sethi et al., 2005; Sonovane et al., 2002). An increase in the number of squares (central and peripheral) crossed and in the number of rearings and reduction in faecal droppings show anxiolytic-like activity in an open field test (Prut and Belzung, 2003). The open field test also displayed that after administration of VVJ (4 mL/kg & 8 mL/kg) and diazepam (1 mg/kg), the number of rearings and the number of squares travelled increased altogether when contrasted with the control group. The increase in open field parameters may be connected with the anxiolytic-like activity of the juice. 

Most of the anxiolytic agents produce their effects by means of potentiation of GABA; by facilitating the opening of GABA intervened chloride ion channels. Activation of GABA_A _receptors exerts an anxiolytic effect. It has additionally been observed that anxiolytic medications could produce their activities through acting on the brain stem reticular system, limbic system and hypothalamus (Christopher, 2002). Flavonoids are famous to have anxiolytic-like activity (Paladini et al., 1999). Quercetin produces anxiolysis by acting on α1β1γ2s GABA_A_ receptors (Goutman et al., 2002). The presence of flavonoids in *V. vinifera* has been reported and also, *V. vinifera* contains quercetin (Kashif et al., 2010). Flavonoids are well known to possess anxiolytic activity. It might be that the anxiolytic-like effect, produced by the juice of *V. vinifera,* is mediated by quercetin and/or other quercetin-like flavonoids.

In conclusion, the present study establishes the anxiolytic-like activity of VVJ in animal models of anxiety. Further studies on the isolated active constituents of the juice of *V. vinifera* and on its mechanism should be conducted hereafter.

## Conflict of interest

The authors declare that there is no conflict of interests regarding the publication of this paper.
